# Microbial metabolite butyrate promotes anti-PD-1 antitumor efficacy by modulating T cell receptor signaling of cytotoxic CD8 T cell

**DOI:** 10.1080/19490976.2023.2249143

**Published:** 2023-08-27

**Authors:** Xinhai Zhu, Ke Li, Guichao Liu, Ruan Wu, Yan Zhang, Siying Wang, Meng Xu, Ligong Lu, Peng Li

**Affiliations:** a Department of Oncology, First Affiliated Hospital, Jinan University, Guangzhou, China; b Department of Geriatrics, The Seventh Affiliated Hospital, Sun Yat-Sen University, Shenzhen, China; c Department of Head and Neck Breast Radiotherapy, The First People’s Hospital of Foshan City, Foshan, China; d The First Affiliated Hospital, Jinan University, Guangzhou, China; e Center for Disease Control and Prevention, Anhui Provincial Center for Disease Control and Prevention, Hefei, China; f Department of Breast Surgery, First Affiliated Hospital, Jinan University, Guangzhou, China; g Guangdong Provincial Key Laboratory of Tumor Interventional Diagnosis and Treatment, Zhuhai Institute of Translational Medicine, Zhuhai People’s Hospital Affiliated with Jinan University, Zhuhai, China; h The Biomedical Translational Research Institute, Faculty of Medical Science, Jinan University, Guangzhou, China

**Keywords:** Gut microbiota, butyrate, PD-1, T cell receptor, anti-tumor immunity

## Abstract

Recent studies have demonstrated that the antitumor immunity of immune cells can be modulated by gut microbiota and their metabolites. However, the underlying mechanisms remain unclear. Here, we showed that the serum butyric acid level is positively correlated with the expression of programmed cell death-1 (PD-1) on circulating CD8^+^ and Vγ9 Vδ2 (Vδ2^+^) T cells in patients with non-small cell lung cancer (NSCLC). Responder NSCLC patients exhibited higher levels of serum acetic acid, propionic acid, and butyric acid than non-responders. Depletion of the gut microbiota reduces butyrate levels in both feces and serum in tumor-bearing mice. Mechanistically, butyrate increased histone 3 lysine 27 acetylation (H3K27ac) at the promoter region of *Pdcd1* and *Cd28* in human CD8^+^ T cells, thereby promoting the expression of PD-1/CD28 and enhancing the efficacy of anti-PD-1 therapy. Butyrate supplementation promotes the expression of antitumor cytokines in cytotoxic CD8^+^ T cells by modulating the T-cell receptor (TCR) signaling pathway. Collectively, our findings reveal that the metabolite butyrate of the gut microbiota facilitates the efficacy of anti-PD-1 immunotherapy by modulating TCR signaling of cytotoxic CD8 T cells, and is a highly promising therapeutic biomarker for enhancing antitumor immunity.

## Introduction

Cancer immunotherapy has achieved impressive success in the treatment of solid and hematological metastatic malignant tumors.^1–4^ The tumor microenvironment (TME) induces T cell dysfunction by attenuating the signal transduction of the T cell receptor.^5–7^ Immune checkpoint inhibitor (ICI) targeted therapies reinvigorate antitumor immune responses by recovering the function of tumor infiltrating T cells (TILs) such as CD4, CD8, and γδ T cells in the TME.^4,8–12^ However, only a sizable minority of cancer patients respond to ICI therapy. Therefore, improving the infiltration and function of T cells in the TME may increase the efficacy of tumor immunotherapies.

Several studies have suggested an association between the gut microbiome (*Bifidobacterium*, *Akkermansia muciniphila*, *Thetaiotaomicron*, *Fragilis* and *Collinsella aerofaciens*) and ICI immunotherapies.^13–21^ The potential mechanisms by which gut microbiota mediate antitumor immunity are through the activation of specific T cell responses against microbial antigens, which either provide support for tumor-specific immune activation or may cross-react against tumor-specific antigens.^17,22,23^ However, it is still not fully understood how the metabolites of the gut microbiota regulate antitumor immunity and whether they can modulate tumor-infiltrating T cell responses.

Short-chain fatty acids (SCFAs), including acetate (AA), propionate (PA), and butyrate (BA), are bacterial fermentation products of the gut microbiota and range in concentration from 50 to 100 mM in the colonic lumen.^24,25^ Recent reports demonstrate that the antitumor immunity of cytotoxic CD8^+^ T cells can be promoted by the metabolite butyrate of gut microbiota,^26–29^ the mechanisms by which butyrate regulates T cell responses and how they work with ICI remain to be further investigated.

In this study, the serum of cancer patients who responded to anti-PD-1 and chemotherapy showed higher butyric acid abundance than that of non-responders. We demonstrated that treatment with anti-PD-1 and butyrate promoted strong production of IFN-γ and TNF-α in CD8^+^ T cells, resulting in increased antitumor activity and enhanced efficiency of anti-PD-1 immunotherapy. These data indicate that gut microbiota-derived metabolites, such as butyrate, have the potential to optimize immunotherapy in patients with cancer.

## Results

### Serum butyric acid amount is correlated with anti-PD-1 efficacy in NSCLC patients

**Figure 1.**
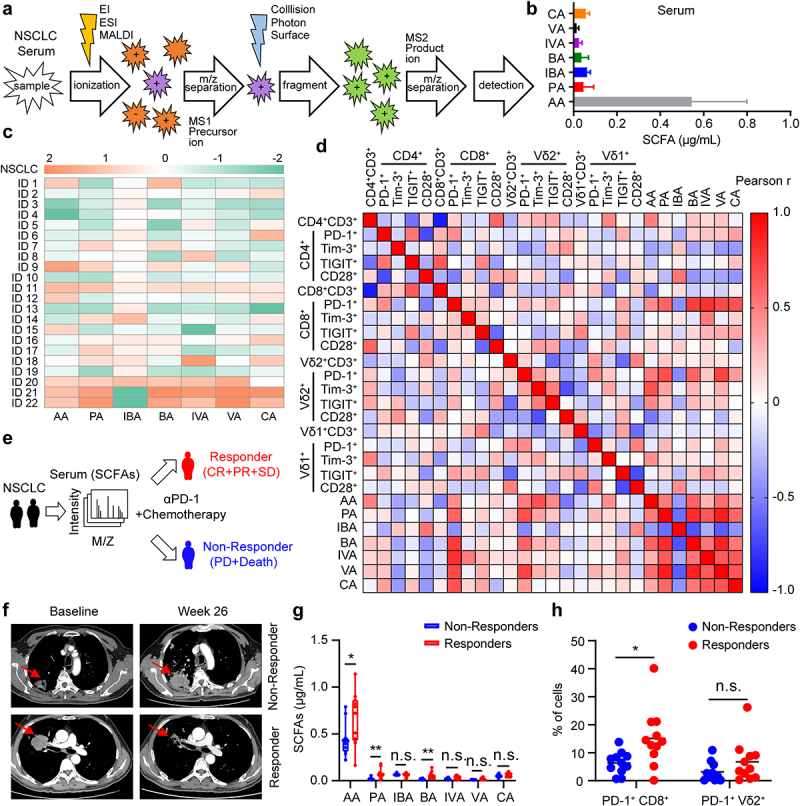
Serum butyric acid amount is correlated with anti-PD-1 efficacy in NSCLC patients. (a) Overview of experimental design. Targeted metabolomics (GC-MS/MS) analysis procedure. (b, c) the serum levels of acetic acid (AA), propionic acid (PA), isobutyric acid (IBA), butyric acid (BA), isovaleric acid (IVA), valeric acid (VA) and caproic acid (CA) in NSCLC patients were detected by GC-MS/MS (*n* = 22). (d) Linear regression analysis between SCFAs (AA, PA, IBA, BA, IVA, VA and CA) levels and surface markers (PD-1^+^, Tim-3^+^, TIGIT^+^, CD28^+^) on T cells (CD4^+^, CD8^+^, Vδ1^+^, and Vδ2^+^) in patients with advanced lung cancer (*n* = 22). (e) experimental design. SCFAs levels in serum of patients with NSCLC were detected by GC-MS/MS before therapy with anti-PD-1 and chemotherapy. (f) CT scans showing changes of tumor size in patients with NSCLC who received intravenous anti-PD-1 and chemotherapy. (g) the serum SCFAs from patients with NSCLC were measured and compared between responders (complete response, partial response or stable disease; CR, PR or SD) and non-responders (progressive disease, PD or death). (h) the level of PD-1 on circulating CD8^+^ and Vδ2^+^ T cells in responders (N) versus non-responders (NR) of NSCLC (N, *n* = 11; NR, *n* = 11). The Pearson correlation was used in (d); two-tailed unpaired Student’s *t*-test (g and h). Data represented mean or mean±SD. *P < .05, **P < .01, ***P < .001, ****P < .0001. n.s., not significant.

Recent studies have demonstrated that the gut microbiota and their metabolites can improve tumor responsiveness to chemo- or immunotherapies.^26,27,30^ However, the underlying mechanism remains unclear. We recruited NSCLC patients to detect SCFA levels in the serum (Figure 1a–c) and surface markers on circulating T cells (Fig. S1a), and a summary of the characteristics of the patients with NSCLC is provided in Supplementary Tables 1 and 2. We found that serum levels of acetic acid (AA), propionic acid (PA), and butyric acid (BA) in NSCLC positively correlated with PD-1 on circulating CD8 and Vδ2 T cells (Figure 1d). Importantly, the serum butyric acid levels in patients with NSCLC were significantly positively correlated with PD-1 on circulating CD8^+^ and Vδ2^+^ T cells (Fig. S1b). We analyzed SCFAs levels in anti-PD-1-treated cancer patients and observed that the serum from patients who responded (complete response + partial response + stable disease) to anti-PD-1 therapy showed higher acetic acid, propionic acid, and butyric acid abundance than that of non-responding patients (Figure 1e–g). Furthermore, we found that the PD-1 level on CD8^+^ T cells in responder NSCLC patients was significantly higher than that in non-responders (Figure 1h). Collectively, these data suggest that serum SCFAs levels, especially butyric acid, may be a biomarker of anti-PD-1 efficacy.

### Depletion of gut microbiota decreases the levels of butyric acid and acetic acid both in fecal and serum of tumor-bearing mice

**Figure 2.**
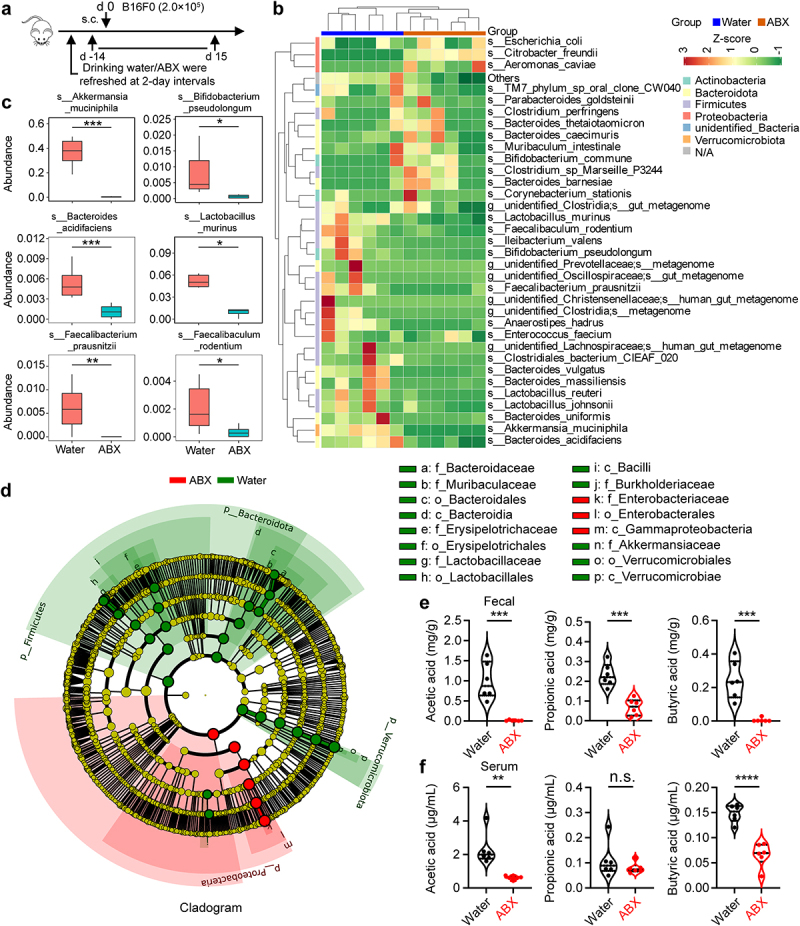
Depletion of gut microbiota decreases the levels of butyric acid and acetic acid both in fecal and serum of tumor-bearing mice. (a) experimental design: mice were treated with antibiotics (ABX, *n* = 6 per group) or drinking water (water, *n* = 6 per group), and inoculated with B16-F0 tumor cells. Feces and serum of the mice were collected on day 15. (b) Cluster heat map of species abundance (p, Phylum; c, class; o, Order; f, family; g, Genus; s, species). (c) relative abundance in water versus ABX of OTU (16S sequencing, based on Metastats analysis). (d) Taxonomic cladogram from LEfSe analysis (*n* = 6). The species without significant difference are uniformly colored yellow, and the difference species biomarker is colored following the group (red=ABX, green=Water). (e, f) targeted metabolomics analysis of gut microbial metabolites (AA, PA, and BA) in colon contents (feces) and serum from mice with or without ABX treatment (*n* = 6 per group). Two-tailed unpaired Student’s *t*-test (c, e, and f). *P < .05, **P < .01, ***P < .001, ****P < .0001. n.s., not significant.

To determine whether the levels of SCFAs in the feces and serum of tumor-bearing mice were associated with the composition of the commensal microbiota, we performed 16S rRNA sequencing combined with targeted metabolomics analyses with samples from the colonic contents and serum of tumor-bearing mice with or without an antibiotic cocktail (ABX) treatment (Figure 2a). Hierarchical clustering of samples based on the relative abundance of OTUs revealed that the gut microbiota, such as *Akkermansia muciniphila*, *Bifidobacterium pseudolongum*, *Bacteroides acidifaciens*, *Lactobacillus murinus*, *Faecalibacterium prausnitzii*, and *Faecalibaculum rodentium* was significantly reduced in ABX-treated mice (Figure 2b,c). To further discuss these findings, we performed high-dimensional class comparisons using linear discriminant analysis of effect size (LEfSe), which again indicated differentially abundant bacteria in the fecal microbiome of control (water) versus ABX, with *Akkermansia muciniphila* species and *Verrucomicrobiales* order enriched in control (water) and *Citrobacter freundii* species enriched in ABX (Figure 2d and Fig. S2a). In addition, antibiotic treatment not only disrupted the composition of gut microbiota but also had a profound impact on gut metabolites, especially SCFAs. Compared with the ABX-untreated mice, the levels of acetic acid and butyric acid in feces and serum were significantly decreased in ABX-treated mice, and were associated with a reduced abundance of *Verrucomicrobiota*, *Firmicutes* and *Bacteroidota* (Fig. S2b, c). To identify the absolute levels of SCFAs, we performed targeted metabolomics (GC-MS/MS) analyses with samples from the colonic contents and serum of tumor-bearing mice with or without an antibiotic cocktail. Indeed, mice in the antibiotic treatment group had lower amounts of acetic acid and butyric acid (Figure 2e,f), with the greatest reduction in acetic acid and butyric acid (Fig. S2d-g). Collectively, these findings suggest that reduced levels of SCFAs in tumor-bearing mice may be the etiology of tumor progression.

### Butyrate supplementation enhances the antitumor immune responses of anti-PD-1

**Figure 3.**
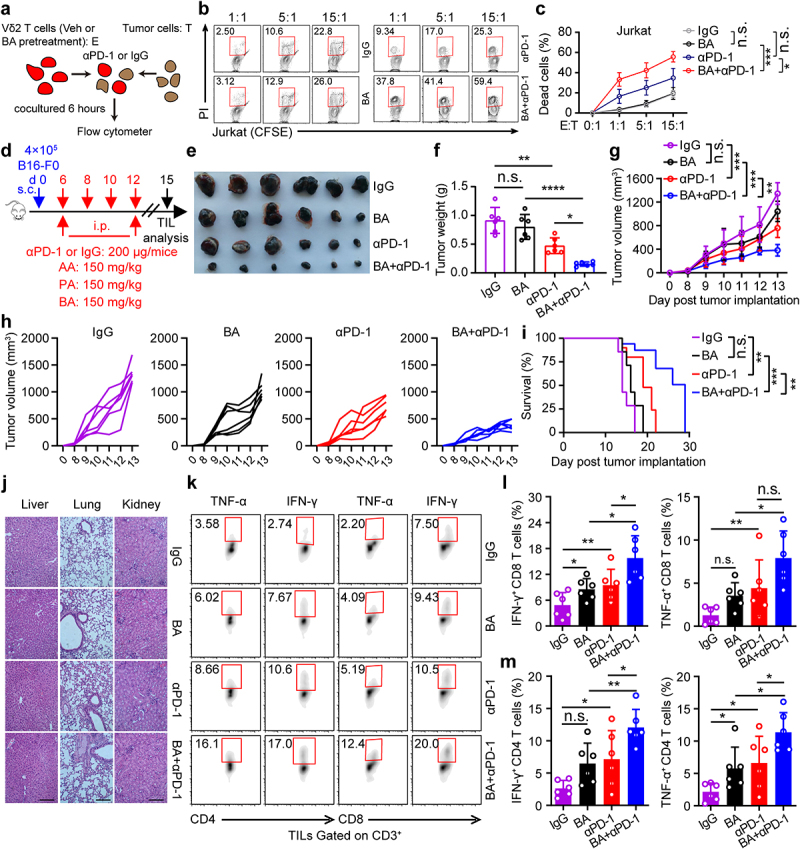
Butyrate supplementation enhances the antitumor immune responses of anti-PD-1. (a) Overview of study design. (b, c) BA pretreated Vδ2 T cells (effector, E) were incubated with Jurkat cells (target, T) at the indicated ratios for 6 hours with or without anti-PD-1 treatment, and the percentages of dead cells out of total target cells identified as PI^+^ were shown (healthy donors, *n* = 3 per group). (d) experiment approach. (e) images of B16-F0 tumors in mice at day 15 post-tumor implantation. (f) tumor weight in the mice in each group at the end of experiment (*n* = 6 per group). (g-i) tumor growth (g and h) and survival curves (i) in the mice after treatment with IgG, butyrate, anti-PD-1 alone or the combination (*n* = 6 per group). (j) Tissue pathology of the indicated organs was evaluated by H&E staining at the end of the experiment (days 15 after B16-F0 tumor implantation). (k-m) tumor-infiltrating IFN-γ^+^ and TNF-α^+^ T cells (CD8^+^ and CD4^+^) were analyzed by flow cytometry (*n* = 6 per group). Two-tailed unpaired Student’s *t*-test used in (c, E:*T* = 15:1); one-way ANOVA with Tukey’s multiple comparisons test (f, g, l and m); log-rank (Mantel-Cox) test was used in (i). Data represented mean±SD. *P < .05, **P < .01, ***P < .001, ****P < .0001. n.s., not significant.

γδ T cells recognize tumor antigens in a major histocompatibility complex-independent manner and can be used as a useful tool for detecting antitumor activity in vitro.^6,31^ We hypothesized that the combination of butyrate and anti-PD-1 might be involved in promoting antitumor immunity in γδ T cells *in vitro*. Zoledronate-expanded Vδ2 T cells accounted for more than 90% of the cultured peripheral blood mononuclear cells (PBMCs) from healthy donors *in vitro* (Fig. S3a), and were mainly effector memory T cell (CD45RA^−^CD27^−^) subpopulations (Fig. S3b). The levels of PD-1 in Vδ2^+^ T cells treated with anti-PD-1 were significantly lower than those in isotype controls (Fig. S3c, d). To validate whether butyrate promoted the therapeutic efficacy of anti-PD-1 in Vδ2 T cells, we performed an *in vitro* tumor-killing experiment (Figure 3a). These results showed that butyrate synergized with anti-PD-1 to facilitate antitumor immunity of Vδ2 T cells in vitro (Figure 3b,c). To further confirm whether SCFAs (acetate, propionate, and butyrate) promoted antitumor immunity of anti-PD-1 *in vivo*, B16F0-bearing mice were intraperitoneally treated with anti-PD-1 alone or in combination with acetate, propionate, or butyrate (Figure 3d). Combination therapy with anti-PD-1 and butyrate resulted in more promising tumor regression (Figure 3e–i). However, in a tumor-bearing mouse model, no obvious difference of tumor size shrinkage was observed under the therapy of anti-PD-1 combined with acetate or propionate compared with that of administration with a single dose of anti-PD-1 (Fig. S3e, f). Hematoxylin-eosin staining showed that tissue injury was not observed following treatment with butyrate or anti-PD-1 (Figure 3j). Interestingly, administration of butyrate and anti-PD-1 increased the percentage of IFN-γ^+^ and TNF-α^+^ in tumor infiltrating CD4^+^ and CD8^+^ T cells compared to treatment with anti-PD-1 or butyrate alone (Figure 3k–m), whereas the expression of antitumor related cytokines in γδ^+^ T cells was either unchanged or only marginally increased (Fig. S3g-i). Taken together, our data showed that butyrate improved the efficacy of anti-PD-1 therapy.

### Microbial metabolite butyrate promotes anti-PD-1 immunotherapy efficacy through CD8^+^ T cell-dependent antitumor immunity

**Figure 4.**
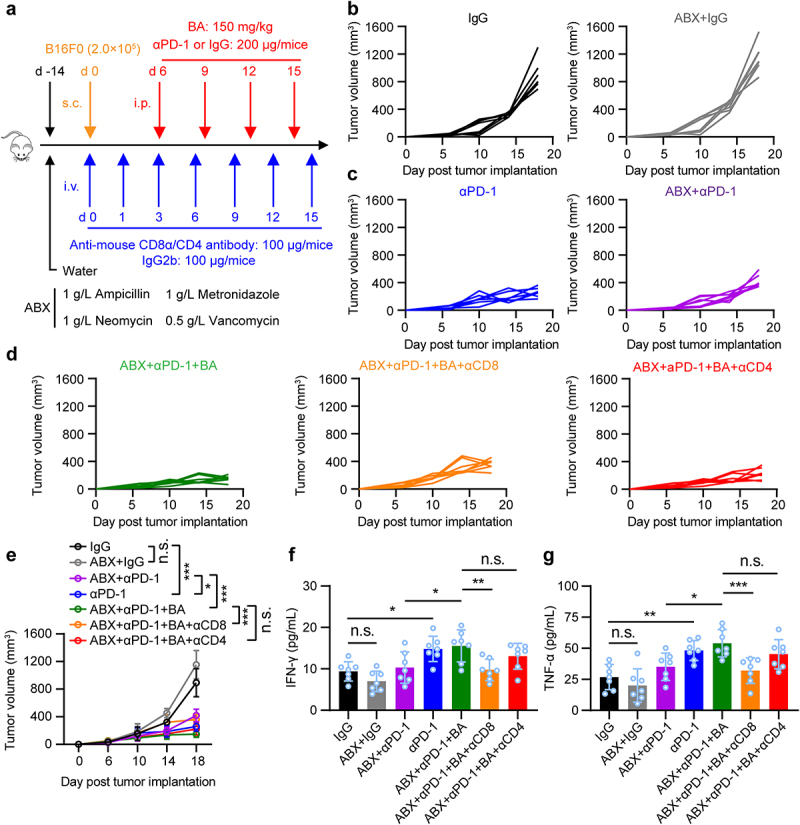
Microbial metabolites butyrate promotes anti-PD-1 immunotherapy efficacy through CD8^+^ T cell-dependent antitumor immunity. (a) experimental design: mice were treated with antibiotics, and inoculated with B16-F0 tumor cells followed by indicated therapy. (b-e) tumor growth was monitored. Mice were inoculated with B16-F0 in ABX pretreated mice, then received anti-PD-1, BA, or anti-PD-1 plus butyrate therapy (*n* = 7 per group). Experiments were independently repeated three times (b-e). (f, g) serum IFN-γ and TNF-α levels in mice were measured by ELISA after indicated treatment (mice serum were collected on day 18), *n* = 7 per group. One-way ANOVA with Tukey’s multiple comparisons test (e-g). Data represented mean±SD. *P < .05, **P < .01, ***P < .001, ****P < .0001. n.s., not significant.

To determine whether butyrate from gut microbiota could promote antitumor immunity of anti-PD-1 through CD8 or CD4 T cells, mice were pretreated with or without an antibiotic cocktail (ABX) of ampicillin, vancomycin, neomycin, and metronidazole in drinking water. Then, B16-F0 melanoma cells were inoculated subcutaneously and treated with anti-mouse CD8/CD4 antibodies to deplete CD8^+^ or CD4^+^ T cells, followed by anti-PD-1, butyrate, or combination treatment (Figure 4a). Importantly, depletion of CD8^+^ T cells, but not CD4^+^ T cells, in ABX-treated B16-F0 tumor-bearing mice dampened the antitumor effect of butyrate combined with anti-PD-1 (Figure 4b–e). Decreased serum IFN-γ and TNF-α production during the depletion of CD8^+^ T cells was also observed (Figure 4f,g), suggesting that anti-PD-1 combined with microbial butyrate treatment could induce IFN-γ and TNF-α production by directly modulating CD8^+^ T cells. Taken together, our data indicate that gut microbiota-derived butyrate could regulate the antitumor response to anti-PD-1 therapy through CD8^+^ T cells.

### Butyrate acts within activated T cells to enhance acetylation of the *Pdcd1* and *Cd28* locus

**Figure 5.**
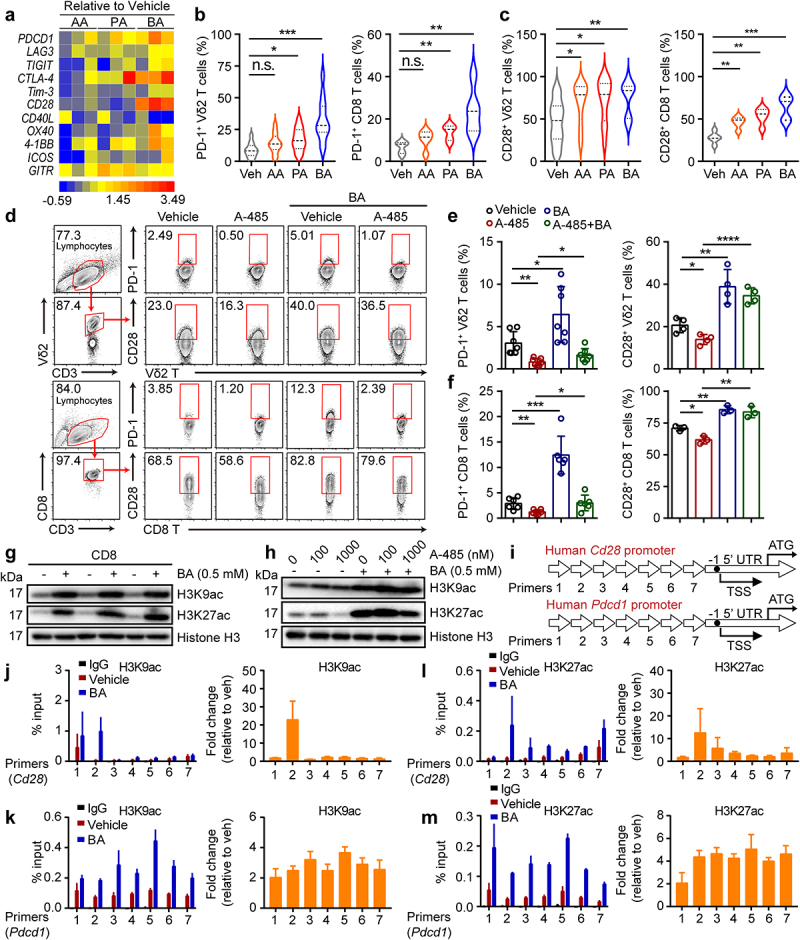
Butyrate acts within activated T cells to enhance acetylation of the *Pdcd1* and *Cd28* locus. (a) mRNA levels of immune checkpoint receptors and co-stimulatory molecules in SCFAs (AA = 10 mM, PA = 1 mM and BA = 0.5 mM) pretreated CD8^+^ T cells were verified by Quantitative real-time PCR (qPCR), and their fold changes were shown in heat map (healthy donors, *n* = 3 per group). (b, c) the expression of PD-1^+^ and CD28^+^ on vehicle, AA, PA, BA-pretreated Vδ2^+^ and CD8^+^ T cells was analyzed by flow cytometry (Vδ2^+^, *n* = 12; CD8^+^, *n* = 5). (d-f) human cytotoxic T cells (CD8^+^ and Vδ2^+^) were treated with BA, A-485 (1 μM) alone or the combination for 48 hours followed by flow cytometry (*n* = 3–7). (g) the levels of histone 3 lysine 9 acetylation (H3K9ac) and histone 3 lysine 27 acetylation (H3K27ac) in vehicle or BA-treated cytotoxic CD8^+^ T cells were determined by immunoblotting. (h) CD8^+^ T cells were treated with or without A485, BA or their combination for 48 hours, followed by western blot. (i-m) Overview of primers design (i). Graph showed ChIP-qPCR analysis of H3K27ac and H3K9ac at the promoter of *Cd28* and *Pdcd1* gene in vehicle or BA-pretreated CD8^+^ T cells. The levels of H3K9ac (j and k) and H3K27ac (l and m) were normalized to the input, *n* = 3 healthy donors. Primers 1–7 (qPCR primers for *Cd28* and *Pdcd1* 1 to 7). Two-tailed unpaired Student’s *t*-test (b and c); one-way ANOVA with Tukey’s multiple comparisons test (e and f). Data represented mean±SD. *P < .05, **P < .01, ***P < .001, ****P < .0001. n.s., not significant.

A previous study reported that the abundance of PD-1 in patients with tumors is a prognostic factor associated with better survival after ICI therapy.^8^ Some studies have demonstrated the CD28-costimulation requirement for CD8 T cell rescue and have suggested an important role for the CD28/B7 pathway in PD-1 therapy in cancer patients.^32,33^ However, it is not yet clear whether butyrate directly regulates the expression of PD-1 and CD28 in T cells. Therefore, we collected a catalog of gut microbial metabolites that were dramatically reduced in ABX-treated mice and screened them for their effects on human Vδ2^+^ and CD8^+^ T cells *in vitro*. Of the SCFAs, butyrate most strongly promoted the expression of PD-1 and CD28 on T cells (Vδ2^+^ and CD8^+^) simultaneously (Figure 5a–c and Fig. S4a), whereas Tim-3 and TIGIT levels in these cells were marginally induced (Fig. S4b-e). In addition, we observed that butyrate significantly induced the expression of PD-L1 in the B16-F0 tumor cell lines whereas the level of PD-L1 was marginally increased by AA or PA (Fig. S4f, g). Other studies have shown that SCFAs are inhibitors of histone deacetylases (HDACs) and ligands for G protein-coupled receptors (GPCRs) and thereby act as signaling molecules that are expressed in an HDAC-dependent manner to regulate T cell homeostasis.^34–36^ How does butyrate promote PD-1 and CD28 expression in CD8^+^ and Vδ2^+^ T cells? To address this, CD8^+^ and Vδ2^+^ T cells were cultured in the presence or absence of a histone acetyltransferase inhibitor (A-485) with or without butyrate treatment. We found that the inhibition of PD-1 and CD28 in response to A-485 was reversed by butyrate (Figure 5d–f), whereas Tim-3 and TIGIT were barely affected (Fig. S4h-k). Consistently, butyrate increased the acetylation of the histone residues H3K9 and H3K27 (Figure 5g). Inhibition of H3K27 acetylation in response to A-485 was reversed by butyrate treatment, while the acetylation of H3K9 was barely changed (Figure 5h). In addition, chromatin immunoprecipitation and quantitative PCR (ChIP-qPCR) analysis confirmed that butyrate enhanced the acetylation of H3K27 and H3K9 in *Cd28* and *Pdcd1* gene promoters (Figure 5i–m). Together, these results revealed that butyrate adopted epigenetic approaches that mediated acetylation of histones to promote the expression of PD-1 and CD28 by human cytotoxic T cells.

### Butyrate regulates the antitumor function of CD8^+^ T cells through T cell receptor signaling

**Figure 6.**
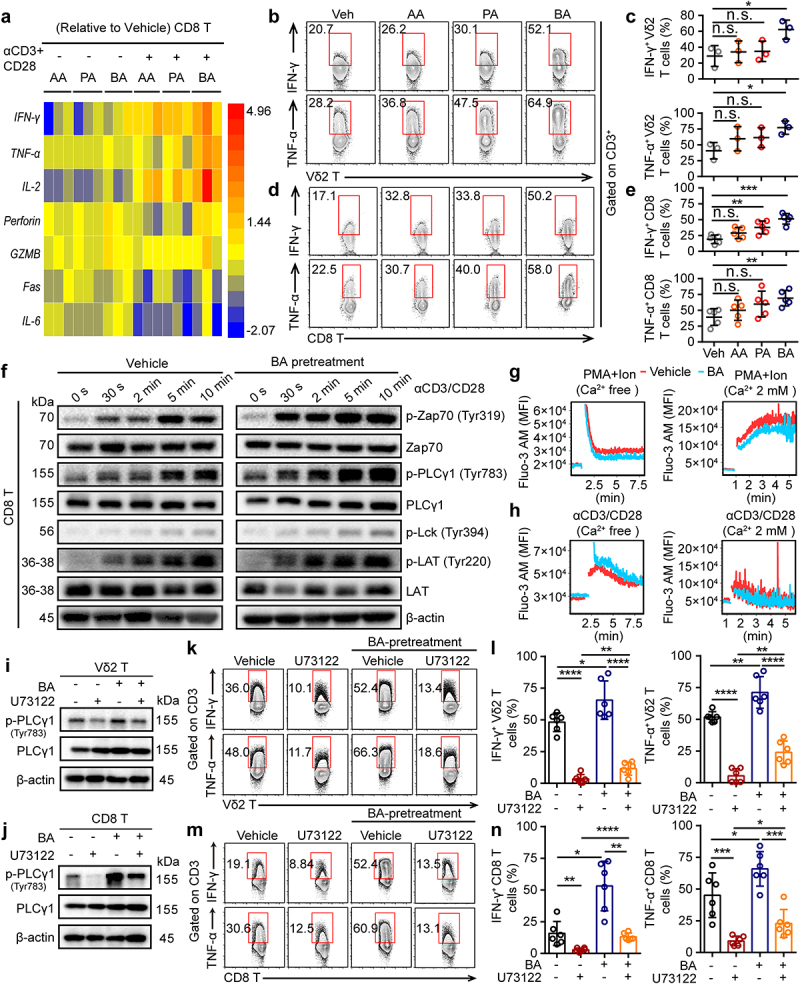
Butyrate regulates the function of CD8^+^ T cells through T cell receptor signaling. (a) vehicle, AA (10 mM), PA (1 mM) or BA (0.5 mM)-pretreated human CD8^+^ T cells were activated with or without α-CD3/CD28 for 4 hours. The expression of various genes related to antitumor immunity in CD8^+^ T cells was measured by qPCR, and their fold changes were shown in heat map (healthy donors, *n* = 3 per group). (b-e) AA, PA, BA or control-pretreated CD8^+^ and Vδ2^+^ T cells were stimulated with α-CD3/CD28 for 4 hours, and the levels of IFN-γ^+^ and TNF-α^+^ in Vδ2^+^ and CD8^+^ T cells were detected by flow cytometry (*n* = 3–5). (f) immunoblotting analysis the phosphorylation of protein from T cell receptor signaling pathway in vehicle or BA-pretreated CD8^+^ T cells upon α-CD3/CD28 activation. (g, h) BA or vehicle-pretreated CD8^+^ T cells were preloaded with fluo-3 AM in Ca^2+^ free medium, then those cells were activated by α-CD3/CD28 or PMA/Ion with or without Ca^2+^ supplementation. Ca^2+^ fluorescence intensity (MFI) transformation was detected by flow cytometry. (i, j) BA-pretreated or vehicle Vδ2^+^ and CD8^+^ T cells were treated with BA, U73122 alone or BA+U73122 for another 1 hour, and then immunoblot analysis the expression of PLCγ1 and p-PLCγ1 from whole-cell lysate. (k-n) BA or vehicle-pretreated Vδ2^+^ and CD8^+^ T cells were treated with or without U73122 under the condition of α-CD3/CD28 activation for 4 hours. IFN-γ^+^ and TNF-α^+^ CD8^+^ or Vδ2^+^ T cells were measured by flow cytometry (*n* = 6). Two-tailed unpaired Student’s *t*-test (c and e); one-way ANOVA with Tukey’s multiple comparisons test (l and n). Data represented mean±SD. *P < .05, **P < .01, ***P < .001, ****P < .0001. n.s., not significant.

To further understand how SCFAs regulate cytotoxic T cells in antitumor responses, CD8^+^ and Vδ2^+^ T cells from healthy donors were cultured for cytokine analyses. The expression of cytokine signature genes in SCFA-pretreated CD8^+^ and Vδ2^+^ T cells was determined using Quantitative Real-time PCR and flow cytometry. Compared with the vehicle (Veh)-pretreated CD8^+^ and Vδ2^+^ T cells, many antitumor-related cytokines were most strongly upregulated in butyrate-pretreated samples, including IFN-γ, TNF-α, and IL-2 (Figure 6a–e and Fig. S5a, b), suggesting that butyrate was required for the activation of cytotoxic T cells upon α-CD3/CD28 stimulation *in vitro*. However, butyrate pretreated CD8^+^ and Vδ2^+^ T cells only treated with phorbol 12-myristate 13-acetate (PMA) and ionomycin (Ion) *in vitro* showed no significant difference in TNF-α, granzyme B (GZMB), and perforin production, whereas the expression of IFN-γ^+^ produced in CD8^+^ was significantly induced (Fig. S5c, d). These results suggest that butyrate may regulate the antitumor function of cytotoxic T cells through the TCR-dependent signaling pathway. To test this hypothesis, butyrate-pretreated CD8^+^ T cells dramatically increased the phosphorylation levels of Lck, Zap70, LAT, and p-PLCγ1, following stimulation with α-CD3/CD28 (Figure 6f). Moreover, TCR activation, but not PMA+Ion, was capable of inducing intracellular Ca^2+^ release from butyrate-pretreated CD8^+^ T cells in Ca^2+^-free medium (Figure 6g,h). A previous report showed that antigen binding to TCR initiates a cascade of protein phosphorylation that converges in the activation of phospholipase Cγ1 (PLCγ1), the production of inositol-1,4,5-trisphosphate (InsP_3_), and Ca^2+^ release from endoplasmic reticulum Ca^2+^ stores via InsP_3_ receptor channels.^37^ Interestingly, the inhibition of PLC-γ1 phosphorylation in response to U73122 was reversed by butyrate treatment in cytotoxic Vδ2^+^ and CD8^+^ T cells (Figure 6i,j). Meanwhile, flow cytometry further confirmed that inhibition of cytokines (IFN-γ^+^ and TNF-α^+^ in Vδ2^+^ T cells; IFN-γ^+^, TNF-α^+^ and GZMB^+^ in CD8^+^ T cells) in response to the PLC-γ1 inhibitor of U73122 was recovered by butyrate, whereas the level of perforin^+^ production in CD8^+^ and Vδ2^+^ T cells was nearly unaffected (Figure 6k–n and Fig. S5e, f). These results demonstrated that butyrate synergized with TCR signaling to enhance the antitumor related-cytokine in cytotoxic CD8^+^ T cells.

## Discussion

Tremendous advances have been made in the treatment of cancer patients using ICIs targeting programmed cell death protein 1 (PD-1), programmed death ligand 1 (PD-L1), and cytotoxic T lymphocyte-associated antigen 4 (CTLA-4).^3,4,8,38^ However, the response rate to ICIs for solid tumors is relatively low. Several studies have indicated that the gut microbiota is a favorable factor associated with the efficacy of ICIs in mouse models and in patients with tumor.^15–17,30^ Upon CTLA-4 blockade, intraepithelial lymphocytes damage ileal epithelial cells, stimulating the accumulation of *Burkholderiales* spp. and *Bacteroides fragilis* and activating IL-12-producing dendritic cells (DCs) and T helper 1 (Th1) immune responses.^16^ Gut microbiota promotes antitumor immunity and facilitates anti-PD-L1 efficacy in tumor-mice model, and one explanation for this finding is that commensal *Bifidobacterium*-derived signals modulate the activation of DCs in the state, which in turn supports improved effector function of tumor-specific CD8^+^ T cells patients.^17^ Similarly, compared with anti-PD-1 immunotherapy responders, non-responders have a reduced abundance of SCFA-producing bacteria, such as *Faecalibacterium* and *Akkermansia muciniphila*.^15,39^

Recent study also demonstrated that in humans, the oxaliplatin responder cancer patients exhibited a higher amount of serum butyrate than did non-responders, which could also increase ID2 expression and function of human CD8^+^ T cells in the absence of anti-PD-1 therapy.^26^ Consistent with previous studies suggesting that butyrate regulates CD8^+^ T cell antitumor immunity,^26,27^ we showed that the microbial metabolite butyrate promotes anti-PD-1 immunotherapy efficacy through CD8^+^ T cell-dependent antitumor immunity. These data suggest that the treatment efficacy of ICI is closely related to adaptive immunity mediated by the commensal microbiome or its metabolites.

Previous studies have shown that SCFAs in mice, especially butyrate, promote the extrathymic generation of Treg cells, induce the differentiation of colonic regulatory T cells, and are regulated by histone deacetylase (HDAC).^34,35,40^ In addition, SCFAs can directly induce T-cell differentiation into T cells producing cytokine, including interleukin-17 (IL-17), IFN-γ, and IL-10, and this effect of SCFAs on T cells is independent of GPR41 (FFAR3) or GPR43 (FFAR2), but dependent on direct HDAC inhibitor activity.^41^ However, several studies have indicated that SCFAs, such as acetic acid, propionic acid, and butyric acid, are major end-product metabolites produced by the gut microbiome and have been confirmed to regulate immune cell response and immunotherapy.^26,27,42^ Our findings showed that the serum butyric acid level is positively correlated with the expression of surface PD-1 on circulating CD8^+^ and Vδ2^+^ T cells in patients with NSCLC. Importantly, responder patients with NSCLC exhibited a higher amount of serum butyric acid than non-responders, suggesting that microbial metabolites might be therapeutically employed. Some studies have revealed that PD-1 suppresses the antitumor function of T cells primarily by inactivating CD28 signaling, indicating an important role for the CD28/B7 pathway in anti-PD-1 therapy in cancer patients.^33,43^ Indeed, we further found that butyrate promotes the expression of CD28 and PD-1 in cytotoxic CD8^+^ and Vδ2^+^ T cells by inducing histone 3 lysine 27 acetylation at the promoters of *Cd28* and *Pdcd1* genes. These results may explain why butyrate improves the efficacy of anti-PD-1 therapy.

T-cell activation requires two signals: a primary antigen-induced signal from the TCR and a secondary signal from co-stimulatory receptors.^44^ Previous reports have shown that the TCR/CD28/Ca^2+^ signaling pathway could enhance the antigen sensitivity of T cells.^37,45^ The present model for TCR activation postulates that after TCR ligation, Lck is activated, which result in phosphorylation of the CD3 coreceptor complex and ζ-chains of the TCR and activation of the ζ-chain-associated protein Zap70, then activated tyrosine kinase Zap70 phosphorylates the membrane adaptor Lat, which subsequently recruits several Src homology – containing proteins, including phospholipase C-γ1 (PLC-γ1).^46–48^ PLC-γ1 regulates multiple pathways to control the production of transcription and inflammatory factors.^47^ Therefore, PLC-γ1 is a central and indispensable molecule in classical TCR signaling. Here, we found that butyrate mediates the phosphorylation of PLC-γ1 and enhances the production of IFN-γ and TNF-α in CD8^+^ T cells under TCR activation. Importantly, the combination of anti-PD-1 and butyrate elicited stronger antitumor immunity responses than a single drug of anti-PD-1 or butyrate in melanoma-bearing mouse models. In addition, the dose of butyrate supplementation in patients with cancer should be determined in the future.

Taken together, our study shows that the gut microbial-derived metabolite butyrate can enhance the efficacy of anti-PD-1 immunotherapy by modulating the T cell receptor signaling of cytotoxic CD8 T cells, suggesting that butyrate is a promising biomarker to sufficiently improve therapeutic efficacy in cancer patients.

## Materials and methods

### Mice

Wild-type mice (C57BL/6J) were obtained from Charles River. All mice were maintained in pathogen-free facilities at Jinan University. All animal experiments were approved by the Institutional Animal Care and Use Committee (IACUC) of Jinan University and complied with all relevant ethical regulations (approval number IACUC-20210706-010). Tumor sizes in all experiments were within the limits permitted by the JNU IACUC.

### Treatment and assessments

Eligible patients had cytologically or histologically confirmed stage III/IV squamous or non-squamous NSCLC without known EGFR sensitizing mutations or ROS1, ALK, or RET fusions, and had no previous systemic treatment for advanced and metastatic disease. Therapy cycles were scheduled according to the clinical trial standard, approved drug interval, and the health condition of each patient. Non-squamous NSCLC patients were received anti-PD-1 (200 mg, intravenously; Sintilimab/Tislelizumab) plus pemetrexed (500 mg/m^2^) and carboplatin (area under the concentration – time curve [AUC] 5 mg/mL per min) intravenously on day 1 of every 3-week cycle for four cycles, followed by anti-PD-1 (200 mg) in combination with pemetrexed (500 mg/m^2^) as maintenance therapy once every 3 weeks for up to 2 years. For squamous NSCLC therapy, patients were received anti-PD-1 (200 mg, intravenously), plus paclitaxel (175 mg/m^2^) and carboplatin (AUC 5 mg/mL per min) intravenously on day 1 of every 3-week cycle, for up to four cycles, followed by maintenance treatment with anti-PD-1 (200 mg). Tumor assessment was performed at baseline, week 6, week 12, and every 9 weeks (2 months) for the first year, and then every 12 weeks until disease progression (PD), loss to follow-up, death, unacceptable toxicity, withdrawal of consent, or study end. The primary outcome of clinical response (responder) was defined by radiographic evidence of complete response (CR), partial response (PR), or stable disease (SD) according to the Response Evaluation Criteria in Solid Tumors version 1.1 (RECIST 1.1) criteria for at least 6 months.^49^ Lack of a clinical response (non-responder) was defined as disease progression (PD) on serial CT scans or stable disease lasting less than 6 months (26 weeks). Information on the healthy donors and patients with NSCLC is provided in Supplementary Tables 1–3.

### Samples analysis

We excluded patients with NSCLC receiving antibiotic treatment, and blood samples from 22 patients, including responders and non-responders, were finally selected for GC/MS/MS and flow cytometry analyses. All experimental protocols, including peripheral blood samples from patients with NSCLC and healthy volunteers, were approved by the Institutional Review Board of the First Affiliated Hospital, Jinan University, Guangzhou, P. R. China (approval number JNU-IRB-KY-2020-10/11). Peripheral blood mononuclear cells (PBMCs) were isolated from patients with NSCLC (before therapy initiation) and healthy donors using the standard Ficoll-Paque-based density gradient centrifugation protocol (GE Healthcare).

### *Human γδ T cell culture* in vitro

Zoledronic acid monohydrate ZOL (MCE, HY-13777A)-expanded Vγ9 Vδ2^+^ (Vδ2^+^) T cells were generated as described below. Human PBMCs (2.5 × 10^6^/mL) were cultured in RPMI 1640 (Gibco, 11875093) medium supplemented with 10% fetal bovine serum (FBS; Gibco, 16140071), 2 mM L-glutamine, antibiotics, and incubated at 37°C in a humidified atmosphere of 5% CO_2_ in air. ZOL (50 μM ZOL and 400 U/mL recombinant human interleukin-2 (rhIL-2; Peprotech, 200-02-50) were added on day 0. RhIL-2 was added at a final concentration of 100 U/mL once every two days from day 3, followed by routine culture procedures for 10 days before further experiments. When ratio of Vδ2^+^ T cells out to total CD3^+^ cells (Vδ2^+^/CD3^+^ T) reached 95%, they could be used in further experiments. ZOL-expanded Vδ2^+^ T cells were further purified by negative selection using the EasySep™ Human Gamma/Delta T Cell Isolation Kit (STEM CELL, 19255).

### Human CD8 T cells isolation

CD8^+^ T cells were isolated with magnetic microbeads (BD, 557766) from PBMCs of healthy donors (purity of CD8^+^/CD3^+^ T cells > 98%), then were stimulated with plate bound 5 μg/mL anti-human CD3 (BioLegend, 300314) and 1 μg/mL anti-human CD28 (BioLegend, 302934) in 48 wells for 48 h. CD8^+^ T cells were cultured in fresh RPMI-1640 medium supplemented with 10% FBS, 100 U/mL rhIL-2, and incubated at 37°C in a humidified atmosphere of 5% CO_2_.

### Cell lines culture

Jurkat and B16-F0 cell lines were obtained from American Type Culture Collection (ATCC, USA). Cell lines were cultured in fresh RPMI 1640 (Jurkat) or DMEM (B16F0) medium supplemented with 10% FBS, 100 U/mL penicillin/streptomycin (Thermo Fisher, 15140122) and incubated at 37°C in a humidified atmosphere of 5% CO_2_.

### In vitro *assays*

For the surface markers and intracellular cytokine detection, CD8^+^ and Vδ2^+^ T cells were treated with 10 mM sodium acetate (AA; Sigma, S2889), 1 mM sodium propionate (PA; Sigma, P1880) or 0.5 mM sodium butyrate (BA; MCE, HY-B0350A) for two times at 2-day intervals. In some experiments, SCFAs pretreated-CD8^+^ and Vδ2^+^ T cells were activated with 5 μg/mL anti-human CD3 and 1 μg/mL anti-human CD28 for 4 h, followed by flow cytometry analysis. For histone acetyltransferase inhibition, CD8^+^ and Vδ2^+^ T cells were treated with control, 0.5 mM BA, 1 μM A-485 (Selleck, S8740), or BA+A-485 for 48 h, and then the expression of PD-1 and CD28 on CD8^+^ and Vδ2^+^ T cells was measured by flow cytometry. Similarly, the levels of histone 3 lysine 9 acetylation (H3K9ac) and histone 3 lysine 27 acetylation (H3K27ac) in protein lysates were detected by immunoblotting. For the p-PLCγ1 detection, vehicle or 0.5 mM BA pretreated CD8^+^ and Vδ2^+^ T cells were treated with or without 2 μM U73122 (Selleck, S8011) under the condition of 5 μg/mL anti-human CD3 and 1 μg/mL anti-human CD28 activation for 1 hour, followed by immunoblotting. Unless mentioned otherwise, SCFAs pretreated T cells (CD8^+^ and Vδ2^+^) used in all the experiments were treated by 10 mM AA, 1 mM PA or 0.5 mM BA for two times at 2-day intervals *in vitro*.

### Immunoblotting

Vehicle- or BA-pretreated T cells (Vδ2^+^, CD8^+^) were lysed in RIPA buffer containing a Protease Inhibitor and Phosphatase Inhibitor Cocktail (Beyotime, P0013K; Bimake, B15001) on ice for 30 min, and the supernatants were used for subsequent analysis. Samples were run on 10% SDS-PAGE gel and then transferred to PVDF membranes, followed by blocking with 5% bovine serum albumin (BSA) for 3 h at room temperature. PVDF membranes were incubated with indicated primary antibodies overnight at 4°C. The following primary antibodies were used: H3K27ac (CST, 8173S), H3K9ac (CST, 9649S), Histone H3 (CST, 4499), p-Lck (CST, 70926S), p-Zap70 (CST, 2717), Zap70 (CST, 2705), LAT (9166), p-LAT (CST, 3584), PLCγ1 (CST, 2822), p-PLCγ1 (14008), PD-L1 (Abcam, ab213480), and β-actin (CST, 3700). The following secondary antibodies were used: HRP-conjugated goat anti-rabbit (CST, 7074) or HRP-conjugated horse anti-mouse (CST, 7076). Immunoblots were visualized using a Bio-Rad ChemiDoc MP Gel Imaging System.

### Cytotoxic assay

Jurkat cells (target) were pre-labeled with 2 μM 5(6)-carboxyfluorescein diacetate succinimidyl ester (CFSE; MCE, HY-D0938). BA pretreated Vδ2^+^ T cells (effector, E) and target cells (T) were coincubated at different E: T ratios (1:1, 5:1, and 15:1) at 37°C in a humidified atmosphere with 5% CO_2_. After 6 h, the cells were harvested and stained with pridium iodide (PI; Sangon Biotech, A601112–0020) for 10 min at room temperature. The percentage of dead cells (CFSE^+^ PI^+^) among the total target cells was identified using flow cytometry. In some experiments, vehicle- or BA-pretreated Vδ2^+^ T cells were treated with or without 10 μg/mL anti-PD-1 (Selleck, A2002) antibody for 30 min, and then these cells were co-incubated with tumor cells at 96 U-shaped wells in the presence of anti-PD-1 treatment for another 6 h. Human IgG4 (10 μg/mL; MCE, HY-P99003) was used as the isotype control for anti-PD-1 treatment.

### Chromatin immunoprecipitation

Vehicle- or BA-pretreated CD8^+^ T cells were subjected to chromatin immunoprecipitation analysis using the Simple ChIP enzymatic chromatin IP Kit (CST, 9003) following the manufacturer’s instructions. Antibodies against H3K27ac (CST, 8173S) and H3K9ac (CST, 9649S) were used. Chromatin-immunoprecipitated DNA was analyzed by RT-qPCR (CFX Connect™ System (Bio-Rad). Primers targeting the promoter within *Pdcd1* and *Cd28* are provided in Supplementary Table S3.

### Mouse tumor models

For the antitumor activity detection, wild type mice (age of 6–8 weeks) were anesthetized with 200 μL 1.25% (w/v) tribromoethanol by intraperitoneal (i.p.) injection. Then, 4.0 × 10^5^ B16-F0 tumor cells were subcutaneously injected into the left flank of mice. When the tumor volume increased to approximately 50–100 mm^3^, mice were intraperitoneally injected with 150 mg/kg AA, 150 mg/kg PA, 150 mg/kg BA, 200 μg/mouse anti-PD-1 (BioXcell, BE0146) alone, or AA/PA/BA combined with anti-PD-1 in 200 μL PBS four times at 2-day intervals. IgG2a (200 μg/mouse; BioXcell, BE0089) was used as an isotype control for anti-PD-1 treatment. For antibiotic cocktail (ABX)-treated mice, 2.0 × 10^5^ B16-F0 melanoma cells were subcutaneously injected into the left flank of mice and treated with anti-CD8 (100 μg/mouse; Selleck, A2102) and anti-CD4 (100 μg/mouse; Selleck, A2101) antibodies via intravenous injection seven times at the indicated time points, while the control groups received the matched isotype control IgG2b (100 μg/mouse; Selleck, A2116) at the same time using the same dose, followed by anti-PD-1 (200 μg/mouse), butyrate (150 mg/kg), or anti-PD-1+butyrate treatment four times at 3-day intervals. Where indicated, mice received a cocktail of antibiotics (0.5 g/L vancomycin, 1 g/L metronidazole, 1 g/L neomycin and 1 g/L ampicillin; MCE) in sterilized drinking water starting 2 weeks before tumor inoculation and continuing until the end of the experiment. The cocktail of antibiotics was supplemented in sterilized drinking water and refreshed every two days throughout the experiment. Tumor volume (TV) was measured by length (L) and width (W) using a Vernier caliper and calculated as tumor volume: $TV=\frac{L\times W^{2}}{2}$. Mice bearing a tumor with size that was larger than 15 mm in any direction were euthanized. All experiments were independently repeated thrice.

### Tumor infiltrating lymphocyte isolation

To analyze the function of tumor-infiltrating T cells (TILs), the mice were euthanized on day 15. Tumor tissues were cut into pieces and suspended in 10 mL tumor digestion buffer (2% FBS, 1.5 mg/mL collagenase IV, and 10 μg/mL DNase I). After rotation for 1 h at 37°C, tumor tissues were digested. The cell suspension was filtered using a 100-μm filter to harvest a single cell. Leukocytes were isolated by density-gradient centrifugation using 40% and 70% Percoll (GE, 17089102). The TILs were then labeled with specific antibodies against the indicated markers. For intracellular staining, TILs were stimulated with 5 μg/mL anti-mouse CD3 (BioLegend, 100340) and 1 μg/mL anti-mouse CD28 (BioLegend, 102116) in the presence of Golgi Stop for 4 h, then fixed and permeabilized with antibodies for another 40 min at 4°C in the dark, all procedures according to the manufacturer’s protocol.

### Flow cytometry

For the flow cytometry analysis, PerCP anti-human TCR Vδ2 (331410), APC anti-human CD27 (356409), PE anti-human CD28 (302907), PE/Cy7 anti-human CD45RA (304125), Pacific Blue™ anti-human CD279 (329915), APC anti-human Tim-3 (345011), PE/Cy7 anti-human TIGIT (372713), PE anti-human TNF-α (502909), FITC anti-human IFN-γ (506504), APC anti-human Perforin (353312), Pacific Blue™ anti-human Granzyme B (515408), APC anti-mouse IFN-γ (505809), FITC anti-mouse TNF-α (506303), PE/Cy7 anti-mouse CD3 (100219), PE anti-mouse CD4 (130310), PerCP/Cyanine5.5 anti-mouse CD8 (140417) and BV421 anti-mouse TCR γδ (118119) were purchased from BioLegend (USA). FITC anti-human TCR Vδ1 (130-100-532) was purchased from Miltenyi Biotec (Germany). V500 anti-human CD3 (561416) antibody was purchased from BD Biosciences (USA). For surface staining, cells were incubated with antibodies for 20 min at 4°C in the dark and then were washed with PBS. For intracellular cytokine staining, cells were pretreated with anti-mouse/human CD3 (5 μg/mL), anti-mouse/human CD28 (1 μg/mL), or PMA (50 ng/mL; Sigma, P8139) plus Ion (1 μg/mL; Sigma, I9657) in the presence of Golgi Stop (BD Biosciences, 554724) for 4 h, then fixed and permeabilized with antibodies for another 40 min at 4°C in the dark, all procedures according to the manufacturer’s recommendations (BD Biosciences, 554715). Flow cytometry was performed using a BD FACS Verse, and data were analyzed using FlowJo software (FLOWJO, V.10.).

### Quantitative real-time PCR

CD8^+^ T cells were collected in 1 mL TRNzol Universal (TIANGEN Biotech, DP424), and RNA was extracted with an RNA Simple Total RNA Kit (TIANGEN Biotech, DP419) according to the manufacturer’s protocol. Approximately 200 ng of RNA was reverse-transcribed using the PrimeScript RT Reagent Kit (TaKaRa, RRO37A). The cDNA was diluted 1:10 in RNase/DNAse-free water for quantitative real-time PCR (qRT-PCR) using a CFX Connect System (Bio-Rad). The relative mRNA expression was calculated using 2^−ΔΔCt^ method. The heat map was described as a fold change, according to the following formula: $Foldchange=\log_{2}^{2^{-\left(Ct_{Target}-Ct_{reference}\right)}}$. qRT-PCR primer pairs were purchased from Sangon Biotech. Primer sequences are provided in Supplementary Table S4.

### Calcium-flux analysis

For intracellular Ca^2+^ detection, approximately 1.0 × 10^6^ CD8^+^ T cells were preloaded with 2 μM Fluo-3 AM (Beyotime, S1056) in Ca^2+^-free medium for 30 min at 37°C, then CD8^+^ T cells were washed twice using Ca^2+^-free medium. Baseline measurements were obtained from cells without activation for 60 seconds (s). At 60 s, 10 μg/mL anti-human CD3 and 1 μg/mL anti-human CD28 or 1 μg/mL Ion plus 50 ng/mL PMA were added. CD8^+^ T cells were harvested using flow cytometry. In some experiments, Ca^2+^-free medium was supplemented with 1.5 mM Ca^2+^, anti-human CD3 (10 μg/mL)/CD28 (1 μg/mL), Ion (1 μg/mL), PMA (50 ng/mL), and Ca^2+^ influx, indicated as fluo-3 AM, was analyzed by flow cytometry.

### Enzyme-linked immunosorbent assay (ELISA)

Serum was collected from the tumor-bearing mice on day 18 to detect the levels of TNF-α and IFN-γ. Briefly, the concentrations of TNF-α (Jianglai Biotech, JL10484) and IFN-γ (Jianglai Biotech, JL10967) were measured using ELISA kits, in accordance with the manufacturer’s instructions (Jianglai Biotech).

### Histology

Mouse livers, kidneys, and lungs were fixed in 4% paraformaldehyde overnight before sectioning. Mouse tissues were stained with hematoxylin and photographed under a microscope. A representative image from each group is shown in our study.

### SCFAs measurements

For serum sample detection, serum samples from NSCLC patients and mice were thawed and vortexed for 1.5 minutes prior to analysis. Serum (50 μL) with patients were supplemented with 0.1 mL of phosphoric acid (36%, v/v) solution in 1.5 mL EP tube, and then the mixture was vortexed for 3 min. 0.15 mL methyl tert-butyl ether (MTBE; CNW Technologies, containing an internal standard) was added. The mixture was vortexed for 3 min and ultrasonicated for 5 min. After that, the mixture was centrifuged at 12,000 rpm/min for 10 min at 4°C. The supernatant was collected and subjected to GC-MS/MS analysis. For fecal sample detection, 20 mg of fecal sample was accurately weighed and placed in a 2 mL tube. 1 mL A phosphoric acid (0.5% v/v) solution and a small steel ball were added to the tube. The samples were uniformly ground, vortexed for 10 min, and ultrasonicated for 5 min. 0.1 mL of supernatant was added to 1.5 mL centrifugal tube after the mixture was centrifuged at 12,000 r/min for 10 min at the temperature of 4°C. 0.5 mL of MTBE (containing an internal standard) solution was added to the centrifuge tube. The mixture was vortexed for 3 min and ultrasonicated for 5 min. After that, the mixture was centrifuged at 12,000 rpm/min for 10 min at the temperature of 4°C. Agilent 8890 gas chromatograph coupled to a 7000D mass spectrometer with a DB-FFAP column (30 m length × 0.25 mm i.d. ×0.25 μm film thickness, J&W Scientific) was employed for GC – MS/MS analysis. SCFAs content was detected using the Agilent 8890–7000D GC-MS/MS platform.

### 16S rRNA sequencing

Total genomic DNA was extracted from the fecal samples using the CTAB method. DNA concentration and purity were determined using 1% agarose gels. The DNA samples were diluted with sterile water to 1 ng/μL. The V3-V4 region of the 16S rRNA gene was amplified using specific primers (515 forward primer: 5’-GTGCCAGCMGCCGCGGTAA-3’ and 806 reverse primer: 5’-GGACTACHVGGGTWTCTAAT-3’). Amplification was performed under the following conditions: initial denaturation at 98°C for 1 min, followed by 30 cycles of denaturation at 98°C for 10 s, annealing at 50°C for 30 s, and elongation at 72°C for 30 s, and finally, 72°C for 5 min. The PCR products were purified using a Qiagen Gel Extraction Kit (Qiagen, Germany). Sequencing libraries were generated using a TruSeq® DNA PCR-Free Sample Preparation Kit (Illumina, USA). Library quality was assessed on a Qubit@ 2.0 Fluorometer (Thermo Scientific) and Agilent Bioanalyzer 2100 system. The 16S rRNA sequencing project was conducted using the Illumina NovaSeq platform.

### Statistical analysis

Statistical analyses were performed using GraphPad Prism 9.0 (GraphPad Software). Data are presented as the mean ± standard deviation (SD). Data were obtained from biologically independent samples. *P* < .05 was considered significant (*P < .05, **P < .01, ***P < .001, and ****P < .0001). All *P* values are indicated in the figures and figure legends.

## Supplementary Material

Supplemental Material

## Data Availability

The 16S rRNA sequencing data that support the findings of this study, are freely available in NCBI SRA at http://www.ncbi.nlm.nih.gov/bioproject/977621 (Bioproject ID: PRJNA977621). All other data supporting the findings of this study are presented in the paper and/or Supplementary Materials. Further inquiries can be directed to the corresponding author.
