# Supplementary material for: Microbial metabolite butyrate promotes anti-PD-1 antitumor efficacy by modulating T cell receptor signaling of cytotoxic CD8 T cell
Source: Gut Microbes. 2023 Aug 27;15(2):2249143. doi: 10.1080/19490976.2023.2249143 (PMC10464552; doi:10.1080/19490976.2023.2249143)
Supplement: Supplemental Material [file KGMI_A_2249143_SM0559.zip › Supplementary tables and figures/Supplementary Figure legends clean.docx]

**Supplementary Figure legends**

**Figure S1. Serum levels of butyric acid in NSCLC were positively correlated with PD-1 on circulating CD8^+^ and Vδ2^+^ T cells.** (**a**) Gating strategy of leukocytes. Immune cells in PBMCs from NSCLC patients were gated by FSC-A/SSC-A to exclude debris, followed by gating CD3^+^ to obtain T cells. (**b**) Serum butyrate level in NSCLC was detected by GC-MS/MS. Linear regression analysis between BA levels and PD-1 on T cells (CD8^+^, Vδ2^+^ and Vδ1^+^). The Pearson correlation was used in (b).

**Figure S2. ABX-treated mice decreased the levels SCFAs both in fecal and serum** (**a**) The LDA score computed for differentially abundant taxa in the gut microbiota of mice in Water and ABX groups (*n*=6 per group). The criteria for feature selection were log LDA score > 4.0. (**b**, **c**) Spearman correlation analysis between differential metabolites (AA, PA, IBA, BA, IVA, and VA from serum and fecal) and relative abundance of differentially abundant taxa in gut microbiota. (**d**, **e**) Targeted metabolomics analysis of gut microbial metabolites (IBA, IVA, VA and CA) in colon contents (feces) and serum from mice with or without ABX treatment (*n*=6 per group). (**f**, **g**) Targeted metabolomics showed the increased gut microbiota metabolites (Water relative to ABX), *n*=6 per group. The Pearson correlation was used in (b and c). Two-tailed unpaired Student’s t-test (d and e). **P*<0.05, ***P*<0.01, ****P*<0.001, **** *P*<0.0001. n.s., not significant.

**Figure S3. The expression of tumor-infiltrating IFN-γ^+^ and TNF-α^+^ γδ^+^ T cells from mice was marginally induced by combination of BA and α-PD-1 therapy.** (**a**, **b**) The subpopulation of Vδ2^+^ T cells were analysed by flow cytometry after vehicle, AA, PA and BA treatment. According to CD27 and CD45RA expression, Vδ2^+^ T lymphocytes were subdivided into: CM (CD27^+^CD45RA^−^), TEMRA (CD27^−^CD45RA^+^), Naïve (CD27^+^CD45RA^+^), and EM (CD27^−^CD45RA^−^). (**c**, **d**) Vδ2^+^ T cells were blockaded by α-PD-1 (0, 1, 5, and 10 μg/mL) or isotype antibody for 0.5 hours *in vitro*. The level of PD-1 was determined by flow cytometry after α-PD-1 treatment. (**e**, **f**) Tumor size (*n*=5 mice per group). Tumor-bearing mice were intraperitoneally injected with 150 mg/kg AA, 150 mg/kg PA, 150 mg/kg BA, anti-PD-1 alone, or AA/PA/BA+anti-PD-1 in 200 μL PBS four times at 2-day intervals. (**g-i**) Tumor-infiltrating IFN-γ^+^ and TNF-α^+^ γδ^+^ T cells were analyzed by flow cytometry. One-way ANOVA with Tukey's multiple comparisons test (e, h, and i). Data represented mean±SD. **P*<0.05, ***P*<0.01, ****P*<0.001, **** *P*<0.0001. n.s., not significant.

**Figure S4. SCFAs did not increase the expression of Tim-3 and TIGIT on cytotoxic CD8^+^ T and Vδ2^+^ T cells *in vitro*.** (**a**) Surface markers on vehicle, AA, PA or BA-pretreated cytotoxic Vδ2^+^ and CD8^+^ T cells were measured by flow cytometry. (**b-e**) The percentage of Tim-3 and TIGIT on vehicle, AA, PA or BA-pretreated Vδ2^+^ (b and c) and CD8^+^ T cells (d and e) were detected by flow cytometry (healthy donors, *n*=5-12). (**f, g**) The level of PD-L1 in vehicle and BA-pretreated B16-F0 tumor cells was determined by western blot. B16-F0 tumor cell lines were treated with vehicle, 0.1 mM and 2.5 mM AA, PA and BA respectively for two times at 1-day intervals. (**h-k**) Cytotoxic CD8^+^ and Vδ2^+^ T cells were treated with BA, A-485 (1 μM) alone or their combination for 48 hours followed by flow cytometry (*n*=6-10). Two-tailed unpaired Student’s *t*-test (c and e); one-way ANOVA with Tukey's multiple comparisons test (j and k). Data represented mean±SD. **P*<0.05, ***P*<0.01, ****P*<0.001, **** *P*<0.0001. n.s., not significant.

**Figure S5. Identification of antitumor-related cytokines in T cells are associated with TCR or PMA/Ion activation.** (**a**, **b**) AA, PA, BA or control- pretreated CD8^+^ and Vδ2^+^ T cells were activated with α-CD3/CD28 for 4 hours, and the MFI of IFN-γ^+^ and TNF-α^+^ in cytotoxic Vδ2^+^ and CD8^+^ T cells were detected by flow cytometry. (**c**, **d**) Flow cytometry and statistical analysis of cytokines of BA or control-pretreated Vδ2^+^ and CD8^+^ T cells, after PMA/Ion treatment for 4 hours in vitro (*n*=6). (**e**, **f**) BA or vehicle-pretreated Vδ2^+^ and CD8^+^ T cells were stimulated with α-CD3/CD28 for 4 hours, then Perforin^+^ and GZMB^+^ CD8^+^ or Vδ2^+^ T cells were measured by flow cytometry (*n*=6). Two-tailed unpaired Student’s *t*-test (c and d); one-way ANOVA with Tukey's multiple comparisons test (e and f). Data represented mean or mean±SD. **P*<0.05, ***P*<0.01, ****P*<0.001, **** *P*<0.0001. n.s., not significant.
