# Supplementary material for: Microbial metabolite butyrate promotes anti-PD-1 antitumor efficacy by modulating T cell receptor signaling of cytotoxic CD8 T cell
Source: Gut Microbes. 2023 Aug 27;15(2):2249143. doi: 10.1080/19490976.2023.2249143 (PMC10464552; doi:10.1080/19490976.2023.2249143)
Supplement: Supplemental Material [file KGMI_A_2249143_SM0559.zip › Supplementary tables and figures/Table S1.docx]

**Table S1. Information of NSCLC patients**

| **Information of NSCLC patients** | |
| --- | --- |
| **Characteristic** | |
| Total | 22 |
| Age, median (range) | 57.5 (29-72) |
| **Sex** | |
| Male | 15 |
| Female | 7 |
| **Stage at diagnosis** | |
| III | 3 |
| IV | 19 |
| **Treatment** | |
| Anti-PD-1+Carboplatin+Paclitaxel | 8 |
| Anti-PD-1+Carboplatin+Pemetrexed | 14 |
| **Assess** | |
| CT | 22 |
| **Responder** (CR+PR+SD) | 11 |
| **Non-responder** (PD+Death) | 11 |
