# Supplementary material for: Microbial metabolite butyrate promotes anti-PD-1 antitumor efficacy by modulating T cell receptor signaling of cytotoxic CD8 T cell
Source: Gut Microbes. 2023 Aug 27;15(2):2249143. doi: 10.1080/19490976.2023.2249143 (PMC10464552; doi:10.1080/19490976.2023.2249143)
Supplement: Supplemental Material [file KGMI_A_2249143_SM0559.zip › Supplementary tables and figures/Table S2.docx]

**Table S2. Information of responder and non-responder NSCLC patients**

| **Information of responder and non-responder NSCLC patients** | | |
| --- | --- | --- |
| **Histologic features** | Non-squamous NSCLC (n=14) | Squamous NSCLC (n=8) |
| **Treatment** | Anti-PD-1+Carboplatin+Pemetrexed | Anti-PD-1+Carboplatin+Paclitaxel |
| **Responder** | Number (n=8) | Number (n=3) |
| CR | 0 | 0 |
| PR | 7 | 3 |
| SD | 1 | 0 |
| **Non-Responder** | Number (n=6) | Number (n=5) |
| PD | 6 | 5 |
| Death | 0 | 0 |
| **Sex** | Number (n=14) | Number (n=8) |
| Male | 8 | 7 |
| Female | 6 | 1 |
| **Stage** | Number (n=14) | Number (n=8) |
| Ⅲ | 1 | 2 |
| Ⅳ | 13 | 6 |

NSCLC, non-small cell lung cancer; CR, complete response; PR, partial response; SD, stable disease; PD, progressive disease.
