# Supplementary material for: Microbial metabolite butyrate promotes anti-PD-1 antitumor efficacy by modulating T cell receptor signaling of cytotoxic CD8 T cell
Source: Gut Microbes. 2023 Aug 27;15(2):2249143. doi: 10.1080/19490976.2023.2249143 (PMC10464552; doi:10.1080/19490976.2023.2249143)
Supplement: Supplemental Material [file KGMI_A_2249143_SM0559.zip › Supplementary tables and figures/Table S3.docx]

**Table S3. Characteristics of healthy donors**

| **Healthy donor characteristics** | | | | | |
| --- | --- | --- | --- | --- | --- |
| **Case ID** | **Gender** | **Age** | **Case ID** | **Gender** | **Age** |
| **1** | Female | 60 | **17** | Female | 26 |
| **2** | Female | 54 | **18** | Male | 53 |
| **3** | Female | 50 | **19** | Male | 63 |
| **4** | Female | 30 | **20** | Male | 36 |
| **5** | Female | 29 | **21** | Male | 62 |
| **6** | Female | 53 | **22** | Male | 64 |
| **7** | Male | 43 | **23** | Male | 49 |
| **8** | Male | 29 | **24** | Male | 45 |
| **9** | Male | 55 | **25** | Male | 56 |
| **10** | Male | 62 | **26** | Male | 60 |
| **11** | Male | 46 | **27** | Female | 45 |
| **12** | Male | 63 | **28** | Female | 24 |
| **13** | Male | 76 | **29** | Female | 22 |
| **14** | Male | 53 | **30** | Female | 45 |
| **15** | Female | 38 | **31** | Female | 34 |
| **16** | Female | 31 | **32** | Female | 55 |
