# Supplementary material for: Microbial metabolite butyrate promotes anti-PD-1 antitumor efficacy by modulating T cell receptor signaling of cytotoxic CD8 T cell
Source: Gut Microbes. 2023 Aug 27;15(2):2249143. doi: 10.1080/19490976.2023.2249143 (PMC10464552; doi:10.1080/19490976.2023.2249143)
Supplement: Supplemental Material [file KGMI_A_2249143_SM0559.zip › Supplementary tables and figures/Table S4.docx]

**Table S4. Primers used for quantitative RT-PCR**

| **Primers used for quantitative RT-PCR all primers are listed in 5’ to 3’ sequence** | | |
| --- | --- | --- |
| **Primer name** | **Forward Primer** | **Reverse Primer** |
| Human *IFN-γ* | TGAATGTCCAACGCAAAGCA | CTGGGATGCTCTTCGACCTC |
| Human *Granzyme B* | CCCTGGGAAAACACTCACACA | GCACAACTCAATGGTACTGTCG |
| Human *TNF-α* | CACAGTGAAGTGCTGGCAAC | AGGAAGGCCTAAGGTCCACT |
| Human *Perforin* | GGCTGGACGTGACTCCTAAG | CTGGGTGGAGGCGTTGAAG |
| Human *IL-2* | AACCTCAACTCCTGCCACAA | GCATCCTGGTGAGTTTGGGA |
| Human *IL-6* | TTCGGTCCAGTTGCCTTCTC | TGAGATGCCGTCGAGGATG |
| Human *Fas* | GGACCCTCCTACCTCTGGTT | ACCTGGAGGACAGGGCTTAT |
| Human *Pdcd1* | GGTGACAGAGAGAAGGGCAG | GTGCGCCTGGCTCCTATTG |
| Human *Lag3* | TGACTGGAGACAATGGCGAC | GGATCCAGGTGACCCAAAGG |
| Human *Tigit* | CTGGACAGGAGGAATGGAGC | GCAGTTACCCAGGCTTCTGT |
| Human *CTLA4* | TACCCACCGCCATACTACCT | TGCAAGGATCCAGAGGAGGA |
| Human *Tim-3* | GAATACAGAGCGGAGGTCGG | TCAAACACAGGACAGGCTCC |
| Human *Cd28* | AGGCTGCTCTTGGCTCTCA | ACAACACAGACTTCCACAGCA |
| Human *CD40L* | CCAGCCTCTGCCTAAAGTCC | TGACAAACACCGAAGCACCT |
| Human *OX40* | GCACGTGGTGTAACCTCAGA | CTCCAGGCTTGTAGCTGTCC |
| Human *4-1BB* | CTTCCTCACGCTCCGTTTCT | AATCGGCAGCTACAGCCATC |
| Human *ICOS* | GGGGGCAAATACTCTGCGAT | TGGCATGAGAATGGTCCAAGT |
| Human *GITR* | CACCCAGTTCGGGTTTCTCA | ACATGCACTGACTCCTCAGC |
| H*uman β-actin* | TTCGACAGTCAGCGCATCTTCTT | GCCCAATACGACCAAATCCGTTGA |
| Human ChIP-*Cd28*-S1 | CAGATCAGGAGGGAGGGACA | ACAAACCCCTAGTGTTGTTACC |
| Human ChIP-*Cd28*-S2 | ACCCACATACACAAAACCCC | CGGCCAACTGCTCTCTTCTT |
| Human ChIP-*Cd28*-S3 | GAGCATGAGACACCAAGGGG | ATTCTACGTGCAAGCAGCCA |
| Human ChIP-*Cd28*-S4 | GCTAAATGCTCCAGAGGGCT | GATGGGGACAGGTTGTGTCA |
| Human ChIP-*Cd28*-S5 | GCCCATCATGTAGTGACCGA | ACCACAAGGCATCCTGACTG |
| Human ChIP-*Cd28*-S6 | GCTGGAACCCTAGCCCATC | AACTAAGCCAATTGGAAGACCT |
| Human ChIP-*Cd28*-S7 | GTATCTTAACAAAAGTCCCTTGGAA | ACATAGTGAAACCCCATCTCAAT |
| Human ChIP-*Pdcd-1*-S1 | AGCGAGTAGCAATGTGGAGAG | GCCATGGGAGAGGAGACCTG |
| Human ChIP-*Pdcd-1*-S2 | CTGGCAGGTGGAATTTTGGG | GGCTCTGAGATGGGAGATGC |
| Human ChIP-*Pdcd-1*-S3 | CCACTTATCCACTGCCTGGG | TGTGCTGCCAGACAGCTAAG |
| Human ChIP-*Pdcd-1*-S4 | GCCTCACATCTCTGAGACCC | CCCGGCTCTGAAGGGAAAA |
| Human ChIP-*Pdcd-1*-S5 | GTTTCTAGCCTCGCTTCGGT | GCGTTTGTGAAATGGCTTGCT |
| Human ChIP-*Pdcd-1*-S6 | GAACTGTGGCCATGGTGTGA | GCACAGGTGACCCTACTGAA |
| Human ChIP-*Pdcd-1*-S7 | GAACCTGAGCCCAGAGGGG | GCCTCTTTCCATATCCCGCC |
