# Supplementary figures and images for: Microbial metabolite butyrate promotes anti-PD-1 antitumor efficacy by modulating T cell receptor signaling of cytotoxic CD8 T cell
Source: Gut Microbes. 2023 Aug 27;15(2):2249143. doi: 10.1080/19490976.2023.2249143 (PMC10464552; doi:10.1080/19490976.2023.2249143)

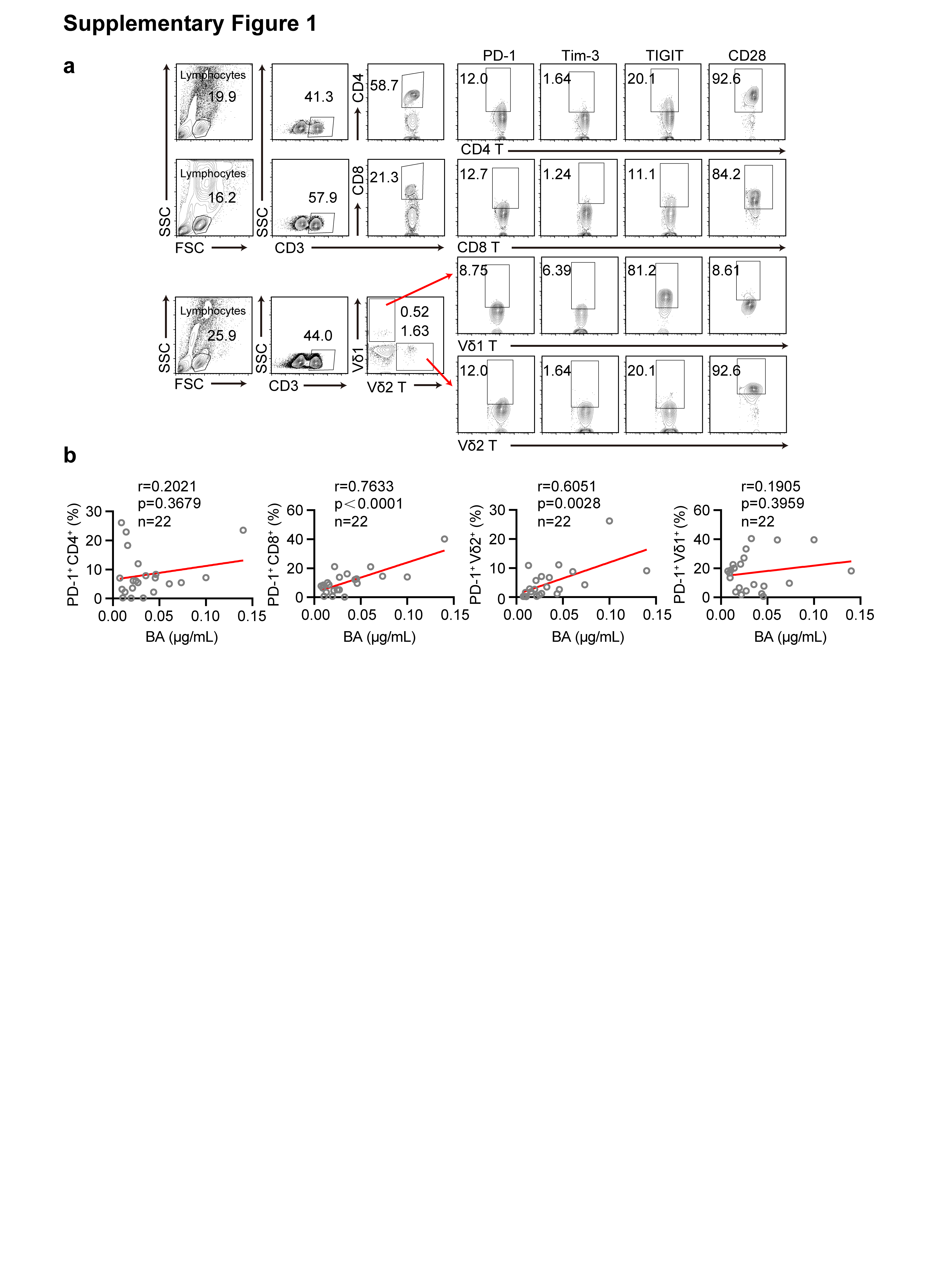

Supplement: Supplemental Material [file KGMI_A_2249143_SM0559.zip › Supplementary tables and figures/Figure S1.tif]

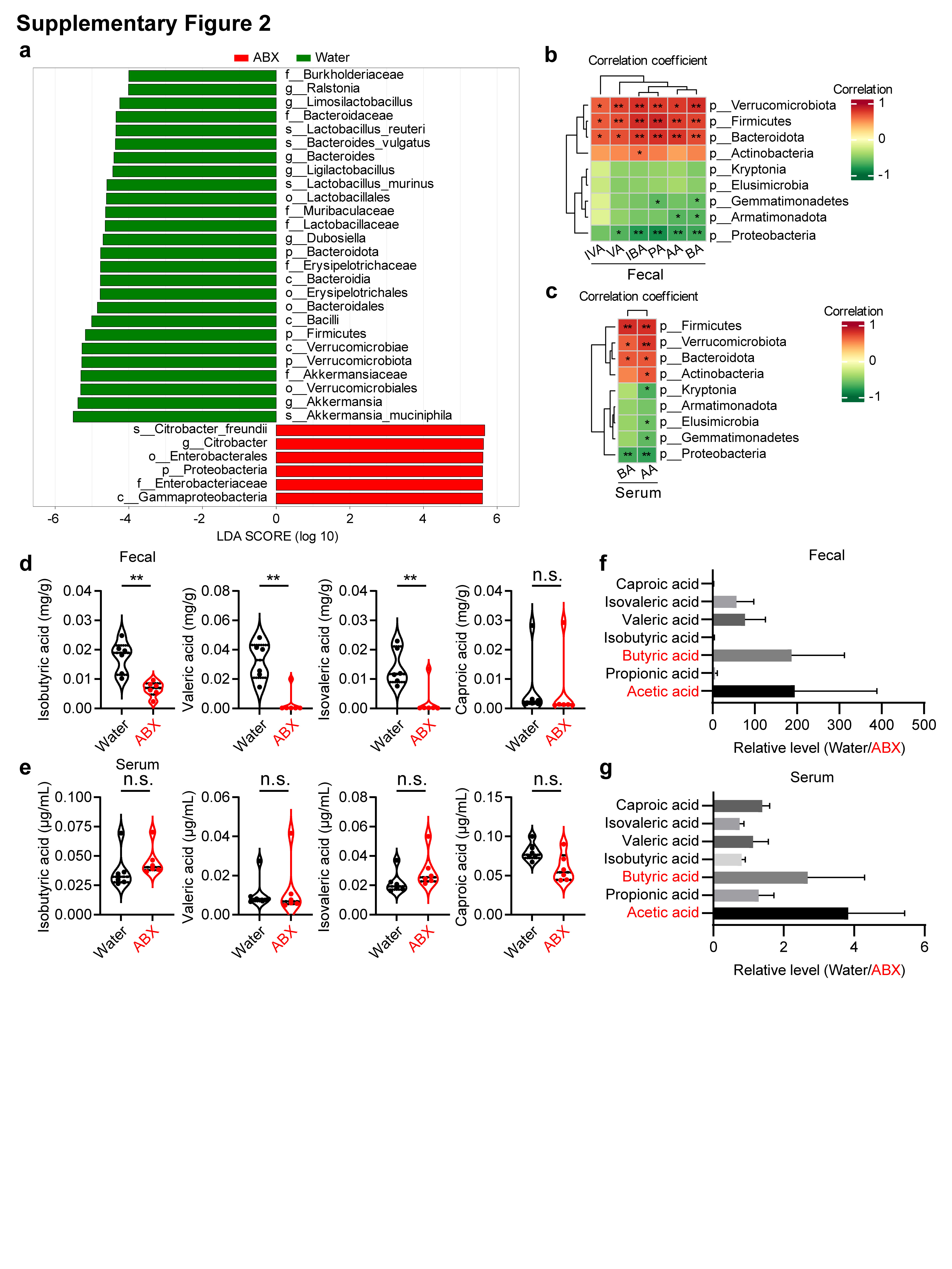

Supplement: Supplemental Material [file KGMI_A_2249143_SM0559.zip › Supplementary tables and figures/Figure S2.tif]

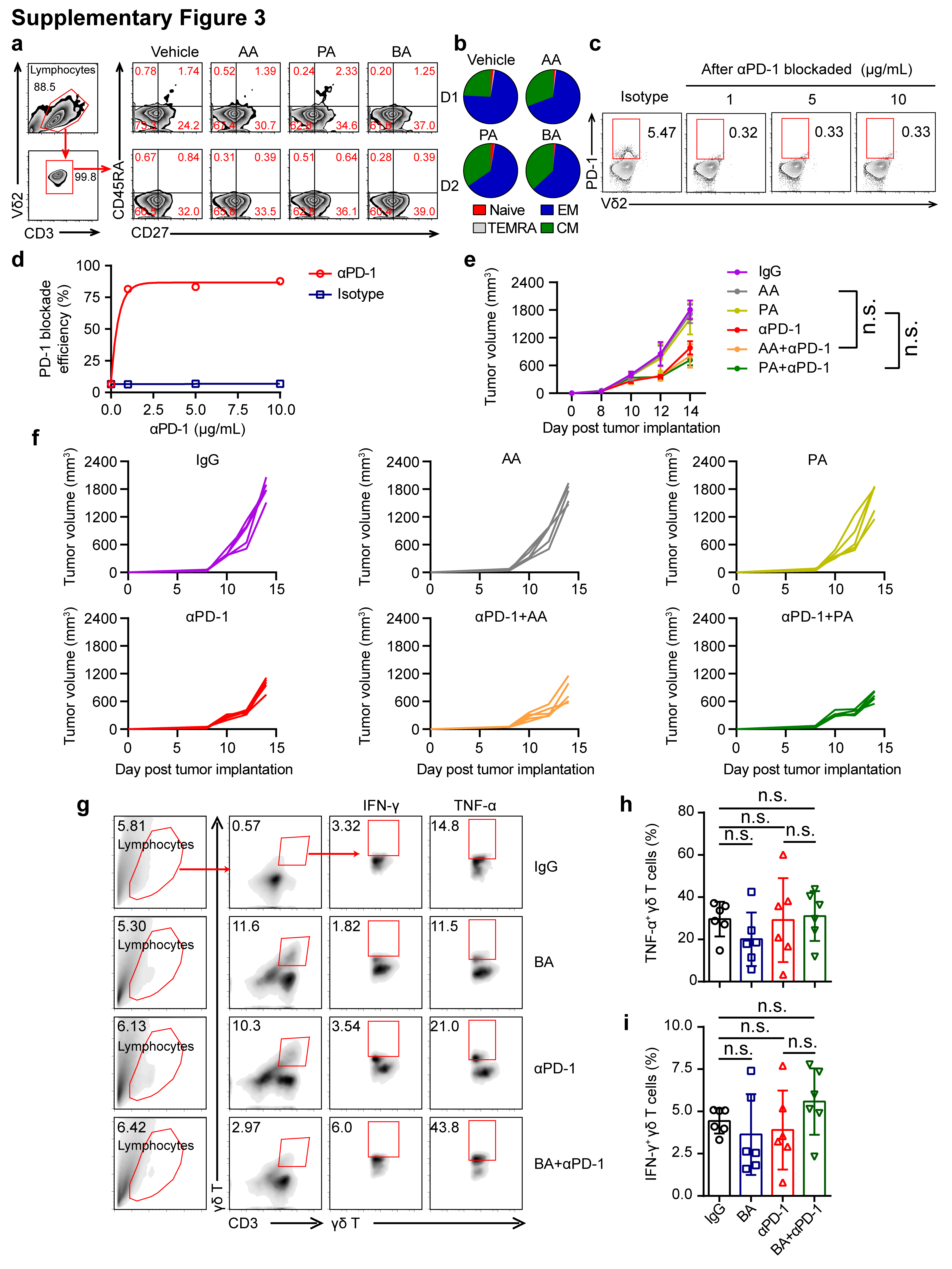

Supplement: Supplemental Material [file KGMI_A_2249143_SM0559.zip › Supplementary tables and figures/Figure S3.tif]

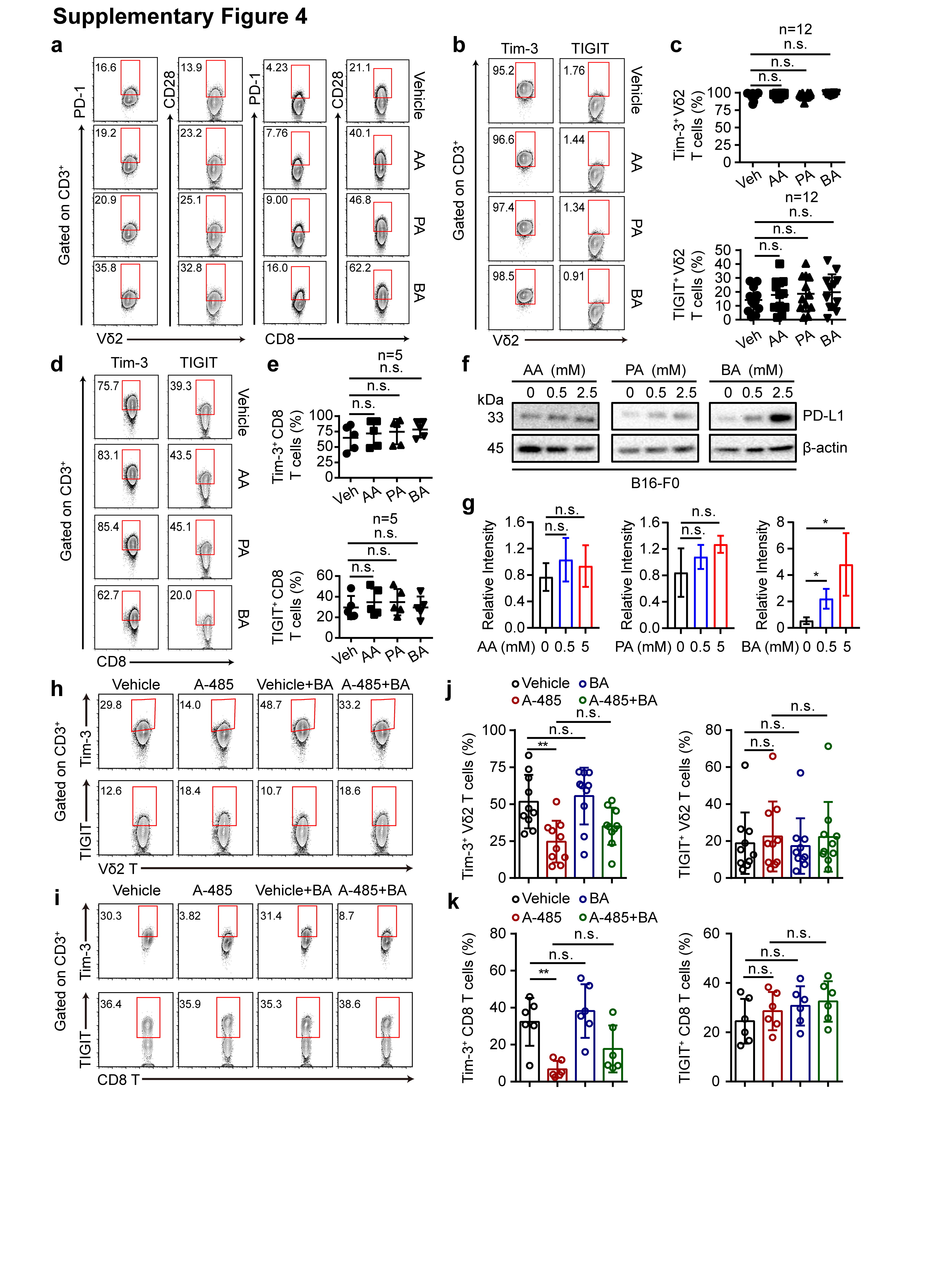

Supplement: Supplemental Material [file KGMI_A_2249143_SM0559.zip › Supplementary tables and figures/Figure S4.tif]

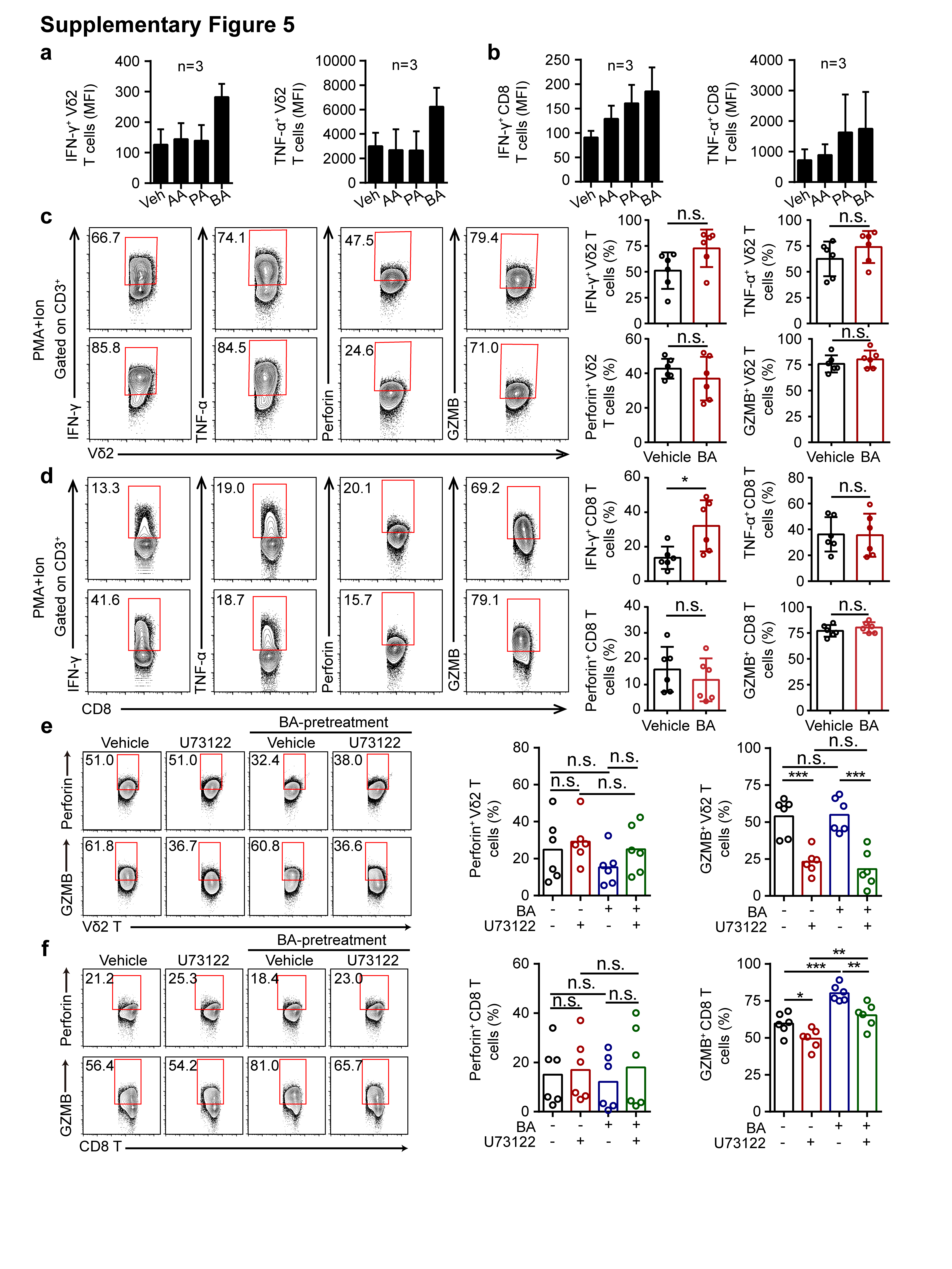

Supplement: Supplemental Material [file KGMI_A_2249143_SM0559.zip › Supplementary tables and figures/Figure S5.tif]
